# Supplementary material for: Quantifying the strength of viral fitness trade-offs between hosts: a meta-analysis of pleiotropic fitness effects
Source: Evol Lett. 2024 Jul 29;8(6):851–65. doi: 10.1093/evlett/qrae038 (PMC11637551; doi:10.1093/evlett/qrae038)
Supplement: qrae038_suppl_Supplementary_material [file qrae038_suppl_supplementary_material.pdf]

Quantifying the strength of viral fitness tradeoffs between hosts: A meta-analysis of pleiotropic fitness effects

Xuechun 'May' Wang[1], Julia Muller[2], Mya McDowell[3] and David A. Rasmussen[1,4,*]

[1] Department of Entomology and Plant Pathology, North Carolina State University, Raleigh, NC, USA.
[2] Gillings School of Global Public Health, University of North Carolina Chapel Hill, Chapel Hill, NC, USA.
[3] Department of Biological Sciences, North Carolina State University, Raleigh, NC, USA.
[4] Bioinformatics Research Center, North Carolina State University, Raleigh, NC, USA.

Supplemental Material

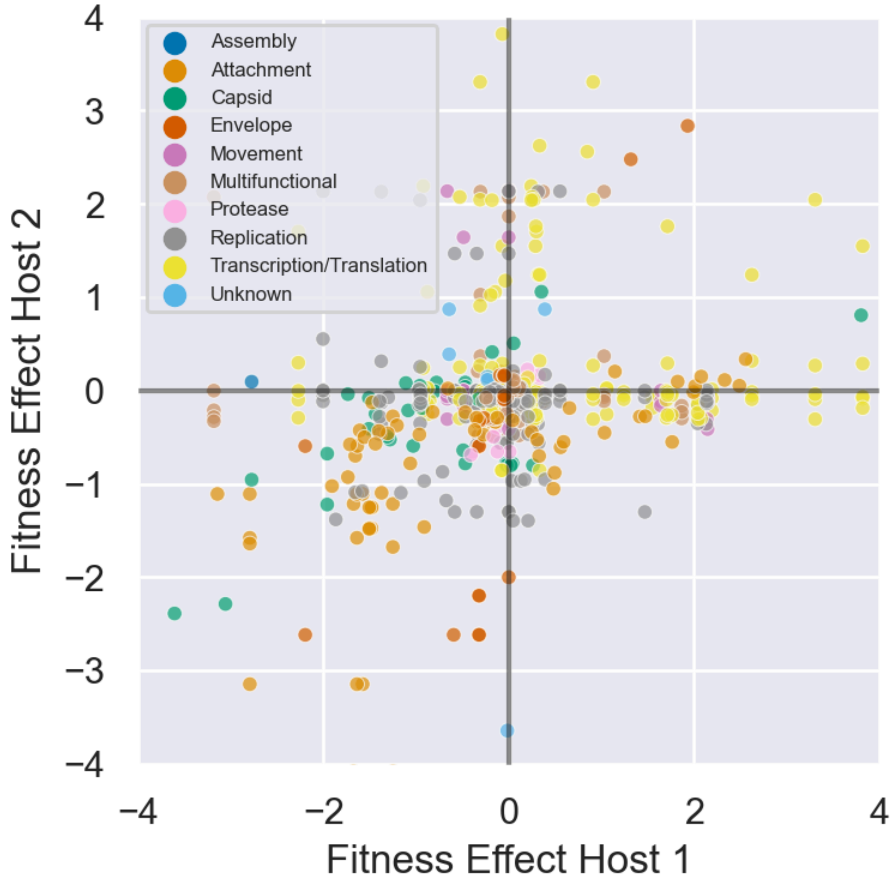

Figure S1: Joint distribution of mutational fitness effects between pairs of hosts colored by the molecular function of the protein in which each mutation occurs.

Table S1. List of the 26 published journal articles used in our meta-analysis.

Authors	Year	Journal	Title	Virus	Hosts	Data source	Quantification method
Pepin et al.	2006	Genetics	Variable Pleiotropic Effects From Mutations at the Same Locus Hamper Prediction of Fitness From a Fitness Component	PhiX174	*Escherichia coli* C (Ec C); *Escherichia coli* (Ec waaL); *Shigella sonnei* (Ss); *Salmonella enterica serovar typhimurium* (St rfb); *Salmonella enterica* (St GalE); *Escherichia coli* (Ec waaV)	Fig.2 and Fig.3	Growth rate (virus titer); Webplot digitizer
Vale et al.	2012	Evolution	The Distribution of Mutational Fitness Effects of Phage φX174 on Different Hosts	PhiX174	*Escherichia coli; Salmonella typhimurium*	Fig. 1	Fitness difference conversion to ratios; Webplot digitizer
Ferris et al.	2007	Genetics	High Frequency of Mutations That Expand the Host Range of an RNA Virus	Phi6	*Pseudomonas syringae* (pathovar phaseolicola); *Pseudomonas syringae* (pathovar japonica)	Contacted author and requested data	Plaque size fitness assay
Hanley et al.	2003	Virology	A Tradeoff in Replication in Mosquito versus Mammalian Systems Conferred by a Point Mutation in the NS4B Protein of Dengue Virus Type 4	Dengue virus	*Chlorocebus sabaeus* (Vero cells); *Homo sapiens* (HuH-7 cells); *Aedes albopictus* (C6/36 cells)	Fig 2	Growth rate; Webplot digitizer
Aytay and	1991	Journal of	Single Amino Acid	Influenza A	*Bos taurus* (MDBK cells);	Table 2	Virus titer

Schulze		Virology	Substitutions in the Hemagglutinin Can Alter the Host Range and Receptor Binding Properties of H1 Strains of Influenza A virus	virus (H1N1)	*Gallus gallus* (CEF cells)		
Scheibner et al.	2023	PLOS Pathogens	Phenotypic Effects of Mutations Observed in the Neuraminidase of Human Origin H5N1 Influenza A Viruses	Influenza A virus (H5N1)	*Gallus gallus* (CEK cells); *Homo sapiens* (NHBE cells)	Fig. 1	Growth rate; Webplot digitizer
Arias Goeta et al.	2014	Infections, Genetics and Evolution	Chikungunya Virus Adaptation to a Mosquito Vector Correlates with only Few Point Mutations in the Viral Envelope Glycoprotein	Chikunguny a virus	*Aedes aegypti; Aedes albopictus*	Fig.2, Fig. 5 and Fig. 6	Virus titer growth rate
Vasilakis et al.	2009	PLOS Pathogens	Mosquitoes Put the Brake on Arbovirus Evolution: Experimental Evolution Reveals Slower Mutation Accumulation in Mosquito than Vertebrate Cells	Dengue virus	*Homo sapiens* (HuH-7 cells); *Aedes albopictus* (C6/36 cells)	Fig. 3 and Fig.5	Growth rate; webplot digitizer
Fitzsimmo ns et al.	2018	PLOS Biology	A Speed Fidelity Trade Off Determines the Mutation Rate and Virulence of an	Poliovirus	*Homo sapiens* (Hela cells); *Mus musculus* (3T3 cells)	Fig.1	Growth rate; webplot digitizer

			RNA Virus				
Coffey and Vignuzzi	2011	Journal of Virology	Host Alternation of Chikungunya Virus Increases Fitness while Restricting Population Diversity and Adaptability to Novel Selective Pressures	Chikunguny a virus	*Homo sapiens* (Hela cells); *Aedes albopictus* (C6/36 cells)	Fig.1	Fitness assays ; webplot digitizer
Greene et al.	2005	Journal of Virology	Effect of Alternating Passage on Adaptation of Sindbis Virus to Vertebrate and Invertebrate Cells	Sindbis virus	*Mesocricetus auratus* (BHK cells); *Aedes albopictus* (C6/36 cells)	Fig.3 and Fig.4	Fitness assays; webplot digitizer
Furio et al.	2012	Journal of Virology	Relationship between within Host Fitness and Virulence in the Vesicular Stomatitis Virus: Correlation with Partial Decoupling	Vesicular stomatitis virus	*Mesocricetus auratus* (BHK21 cells); *Mus musculus*	Table 3 and Table 2	Fitness assays
Van Slyke et al.	2012	Virology	Point Mutations in the West Nile Virus (Flaviviridae; Flavivirus) RNA Dependent RNA Polymerase Alter Viral Fitness in a Host-Dependent Manner in Vitro and in Vivo	West Nile virus	*Chlorocebus sabaeus* (Vero cells); *Aedes albopictus* (C6/36 cells); *Culex tarsalis* (CxT cells); *Gallus gallus* (DF-1 cells); *Gallus gallus*	Fig. 3 and Fig.5	Growth rates; Webplot digitizer
Weaver et al.	1999	Journal of Virology	Genetic and Fitness Changes Accompanying Adaptation of an Arbovirus to Vertebrate and Invertebrate Cells	Eastern equine encephalitis virus	*Mesocricetus auratus* (BHK cells); *Aedes albopictus* (C6/36 cells); *Chlorocebus sabaeus* (Vero cells); *Anopheles albimanus*	Fig.1 and Fig. 2	Virus titer fitness assays; Webplot digitizer

					(pericardial cells)		
Hashemi et al.	2018	Journal of Virology	Mutations in the Non Structural Protein Coding Sequence of Proto Parvovirus H1PV Enhance the Fitness of the Virus and Show Key Benefits Regarding the Transduction Efficiency of Derived Vectors	H1 parvovirus (H1PV)	*Homo sapiens* (NB324K cells); *Homo sapiens* (Hela cells); *Rattus norvegicus* (RG2 cells);	Fig.2	Growth curve; webplot digitizer
Tsetsarkin et al.	2007	PLOS Pathogens	A Single Mutation in Chikungunya Virus affects Vector Specificity and Epidemic Potential	Chikunguny a virus	*Mesocricetus auratus* (BHK21 cells); *Aedes albopictus* (C6/36 cells); *Aedes aegypti; Aedes albopictus*	Fig. S3, Table S2, Table S1, Fig. 6, Fig. S2	Growth rate; webplot digitizer
Obadan et al.	2019	Journal of Virology	Flexibility In Vitro of Amino Acid 226 in the Receptor-Binding Site of an H9 Subtype Influenza A Virus and Its Effect In Vivo on Virus Replication, Tropism, and Transmission	Influenza A virus (H9N1 and H9N2)	*Canis familiaris* (MDCK cells); *Gallus gallus* (DF-1 Cells)	Fig. 3	Growth curve; webplot digitizer
Lemos et al.	2020	Journal of Virology	Two Sides of a Coin: a Zika Virus Mutation Selected in Pregnant Rhesus Macaques Promotes Fetal Infection in Mice but at a Cost of Reduced Fitness in Nonpregnant Macaques	Zika virus	*Aedes albopictus* (C6/36 cells); *Chlorocebus sabaeus* (Vero cells)	Fig. 3	Growth curve; webplot digitizer

			and Diminished Transmissibility by Vectors				
Hillung et al.	2015	Phil Trans B	Evaluating the within Host Fitness Effects of Mutations Fixed during Virus Adaptation to Different Ecotypes of a New Host	Tobacco etch virus	*Arabidopsis thaliana* (Di-2); *Arabidopsis thaliana* (Wt-1); *Arabidopsis thaliana* (Ei-2); *Arabidopsis thaliana* (Ler-0); *Arabidopsis thaliana* (St-0)	Table 3	Fitness assays
Moreno Perez et al.	2016	Journal of Virology	Mutations That Determine Resistance Breaking in a Plant RNA Virus Have Pleiotropic Effects on Its Fitness That Depend on the Host Environment and on the Type, Single or Mixed, of Infection	Pepper mild mottle virus	*Capsicum annuum* cv. Dulce Italiano (L+/L+); *Capsicum annuum* cv. Yolo Wonder (L1/L1); *Capsicum frutescens* cv. Tabasco (L2/L2);	Table 2	Fitness assays
Lalic et al.	2011	PLOS Pathogens	Effect of Host Species on the Distribution of Mutational Fitness Effects for an RNA Virus	Tobacco etch virus	*Nicotiana tabacum; Nicotiana benthamiana; Datura stramonium; Capsicum annuum; Solanum lycopersicum; Helianthus annuus; Gomphrena globosa; Spinacea oleracea*	Fig. 3	Fitness assays
Bera et al.	2018	Journal of Virology	Analysis of Fitness TradeOffs in the Host	Tobacco mild green	*Capsicum annuus* cv. Doux des Landes;	Table 6	Fitness assays

			Range Expansion of an RNA Virus, Tobacco Mild Green Mosaic Virus	mosaic virus	*Nicotiana glauca*		
Moury et al.	2011	Molecular Biology and Evolution	dN/dS Based Methods Detect Positive Selection Linked to Tradeoffs between Different Fitness Traits in the Coat Protein of Potato virus Y	Potato virus Y	*Nicotiana tabacum* cv. Xanthi; *Solanum tuberosum* cv. Bintje	Fig. 2A	Growth rate; webplot digitizer
Cervera et al.,	2016	Journal of Virology	Effects of Host Species on Topography of the Fitness Landscape for a Plant RNA Virus	Tobacco etch virus	*Arabidopsis thaliana; Nicotiana tabacum*	Fig.2	Fitness assays; webplot digitizer
Ayme et al.	2006	Molecular Plant Microbe Interactions	Different Mutations in the GenomeLinked Protein VPg of Potato virus Y Confer Virulence on the pvr23 resistance in pepper	Potato virus Y	*Nicotiana clevelandii; Capsicum spp.* cv. Yolo Wonder	Table 4	Fitness assays
Martinez Turino et al.	2021	Microorgani-sms	Virus Host Jumping Can be Boosted by Adaptation to a Bridge Plant Species	Plum pox virus	*Arabidopsis thaliana; Nicotiana clevelandii*	Fig. 1 and S3	Virus accumulation; Image J

Table S2. Adherence to PRISMA-EcoEvo (version 1.0) guidelines for reporting practices in meta-analyses (O'Dea et al., 2021).

Item No.	Item name	Met criteria?	Explanation
1	Title and abstract	Yes	We mention "meta-analysis" both in the title and abstract.
2	Aims and questions	Yes	We mention our research goals in the introduction and abstract.
3	Review registration	No	We did not register our study's aims or hypotheses before starting our analysis.
4	Eligibility criteria	Yes	We include our literature search criteria in the methodology.
5	Finding studies	Yes	We list all articles our data was sourced from in the supplementary file Table S1. Additionally, we provide our filtering keywords in the methodology.
6	Study selection	Yes	We read all articles our literature initially identified and decided whether to include based on our criteria. Data collection was initially performed by Muller and McDowell. Wang joined and double checked the data and added more datasets. Rasmussen supervised data collection and double checked data.
7	Data collection process	Yes	Where in each study data was extracted from is described in supplementary Table S1. Data collection was performed at the same time when the article was initially reviewed.
8	Data items	Yes	We only sourced the data from published articles, either tables, figures, or supplementary files. We provide additional information on how the data was extracted and analyzed in supplementary Table S1.
9	Assessment of individual study quality	Yes	All studies were reviewed to see if fitness effects were measured using appropriate standardized methods.
10	Effect size measures	Yes	Our effect sizes correspond to the reported fitness effect of each mutation in each hose. How fitness effects were standardized and compared is described in the Methods section.

11	Missing data	Yes	If mutational fitness effects were missing from a particular host, that host was excluded from our analysis.
12	Meta-analytic model description	Yes	The linear and mixed linear regression models we used to analyze how fitness effects vary by mutational, viral and host features while accounting for differences between studies (random effects) are described in the Methods.
13	Software	Yes	All software and packages used to analyze the data are described in the Methods.
14	Non-independence	Yes	Some of the mutational fitness effects may be non-independent e.g. multiple fitness effects from the same study. Likewise fitness effects may be expected to (co-)vary based on the phylogenetic relationships between viruses and hosts. We account for these sources of non-independence by grouping fitness effects by study, the taxonomic family of viruses, and the phylogenetic relationships (i.e. divergence times) among hosts.
15	Meta-regression and model selection	Yes	The moderators or features we included in our regression analysis were chosen based on their importance to viral biology or those known from the literature to impact the distribution of fitness effects. While no formal model selection process was performed, we typically fit several alternative models with and without different features to ensure our overall results were robust to correlations between features.
16	Publication bias and sensitivity analyses	Yes	There is undoubtedly a publication bias resulting in certain viruses or viral families being overrepresented in our analysis. We accounted for these biases by ensuring our main results about the distribution of pleiotropic effects hold more generally and not just for overrepresented taxa. We also performed a sensitivity analysis to ensure our main results about the distribution of pleiotropic effects are robust against varying levels of measurement error in reported fitness effects.
17	Clarification of post hoc analyses	Yes	We explain when certain analyses (e.g. the inclusion of phylogenetic divergence times between hosts) were performed post hoc based on

			prior analysis of the same data.
18	Metadata, data and code	Yes	The extracted fitness effects and associated metadata are all available at: https://github.com/davidrasm/PleiotropyMetaAnalysis
19	Results of study selection process	Yes	We report the number of studies included/excluded.
20	Sample sizes and study characteristics	Yes	The total number of studies, viruses, hosts and mutations are reported in the Results. The number of mutations belonging to each group or subset of viral, host and mutational types are reported in Tables 1, 2, 4 and 5.
21	Meta-analysis	Yes	We report mean fitness/pleiotropic effects as well as regression coefficients quantifying the impact of different features on fitness effects and their associated confidence intervals.
22	Heterogeneity	Yes	The variability in fitness effects due to between-study variation was computed using intraclass correlation coefficients (ICC) values.
23	Meta-regression	Yes	We report regression coefficients and confidence intervals for the effect of features of mutational fitness effects and the magnitude/sign of pleiotropy. These statistics are also reported for interaction effects in our linear models
24	Outcomes of publication bias and sensitivity analyses	Yes	We performed a sensitivity analysis to see how robust our main results are to different levels of simulated measurement error in the reported fitness effects (see Methods).
25	Discussion	Yes	We summarize our main findings and compare these results with previous work. We also discuss limitations including potential publication biases.
26	Contributions and funding	Yes	Author contributions and funding information is provided.
27	References	Yes	We provide a full list of references to all included studies.

Table S3: Mutational fitness effects in single hosts grouped by viral family. Regression coefficients, P-values and 95% CIs are estimated under a mixed effect linear model that included a random effect (intercept) for each study.

Variable	N	Group mean	Estimated coefficient	P-value	95% CI
Intercept	457	-	-0.688	0.65	-1.838 : 0.463
Flaviviridae	34	-0.1	0.589	0.386	-0.743 : 1.920
Microviridae	103	-1.09	-0.425	0.553	-1.830 : 0.980
Orthomyxoviridae	44	0.35	0.624	0.392	-0.805 : 2.054
Parvoviridae	9	0.0001	0.688	0.432	-1.026 : 2.401
Picornaviridae	2	0.0048	0.692	0.512	-1.379 : 2.764
Potyviridae	187	0.16	0.916	0.155	-0.345 : 2.177
Rhaboviridae	2	-1.59	-0.903	0.393	-2.975 : 1.169
Togaviridae	28	0.24	1.184	0.078	-0.132 : 2.501
Virgaviridae	12	-0.23	0.33	0.669	-1.183 : 1.843

Table S4: Sensitivity of the distribution of pleiotropic effects to measurement error in reported fitness effects. Different levels of error were simulated by adding error terms sampled from a Normal distribution with varying standard deviation σ to the observed fitness effects. Fraction +/- refers to all mutations with antagonistic effects between hosts whereas fraction +/+ refers to the fraction of mutations with unconditionally beneficial effects. The χ^2 statistic corresponds to the test for the independence of the sign of fitness effects between hosts. Reported statistics represent the mean of 1,000 simulations performed at each level of error (σ).

Error std dev	Corr coeff	Fraction +/-	Fraction +/+	χ^2	χ^2 p-value
0	0.32	0.43	0.14	7.19	0.126
0.01	0.32	0.41	0.13	8.59	0.085
0.05	0.32	0.42	0.14	8.85	0.095
0.1	0.32	0.43	0.16	9.24	0.102
0.25	0.31	0.44	0.19	9.33	0.124
0.5	0.27	0.45	0.21	8.38	0.172
1	0.18	0.47	0.23	6.13	0.296
